# Effect of circulating anti-Mullerian hormone on the reproductive potential of gilts

**DOI:** 10.3389/fvets.2025.1454343

**Published:** 2025-02-27

**Authors:** Wei Xia, Zhenmin Zhou, Linghua Cheng, Xiaohuan Fang, Chenyu Tao, Yatian Qi, Yang Yu, Di Zhang, Xiaofeng Tian, Zihao Gao, Jiahua Bai, Junhui Wen, Yan Liu, Junjie Li

**Affiliations:** ^1^College of Animal Science and Technology, Hebei Agricultural University, Baoding, China; ^2^Hebei Technology Innovation Center of Cattle and Sheep Embryo, Baoding, China; ^3^Beijing Dabeinong Technology Group, Beijing, China; ^4^Institute of Animal Husbandry and Veterinary Medicine, Beijing Academy of Agriculture and Forestry Sciences, Beijing, China

**Keywords:** anti-Mullerian hormone, puberty, reproductive performance, retention rate, gilt

## Abstract

**Introduction:**

Determination of the ovarian follicular reserve is of great value for predicting fertility, while circulating anti-Müllerian hormone (AMH) plays an important role in maintaining the ovarian reserve.

**Methods:**

In this study, we examined the effect of circulating AMH levels of gilts between 110 and 160 days of age on reproductive performance and evaluated the differences in the ovaries and uteruses of gilts with different AMH levels.

**Results:**

The results indicated a significant negative correlation between circulating AMH levels from days 110 to 160 and puberty (*p* < 0.05). Moreover, the total born, live born, and the herd retention rate across 3 successive parities were found to be higher in high-AMH gilts than in low-AMH ones. Uterine morphology was assessed, and it was found that high-AMH gilts had significantly increased uterine glandular density, and the median vascular area and the relative expression levels of *FOXA2*, *VEGF*, *VEGFR,* and *CD31* were significantly increased (*p* < 0.05). Furthermore, high-AMH gilts had a greater number of antral follicles and higher expression levels in secondary and early antral follicles than low-AMH gilts (*p* < 0.05). To further explain this mechanism, RNA-seq analysis was performed, which indicated that differentially expressed genes (DEGs) of high-AMH gilt ovaries were enriched in pathways, including steroid hormone biosynthesis, arachidonic acid metabolism, and the gonadotropin-releasing hormone signaling pathway.

**Discussion:**

Our findings indicate that circulating AMH levels can possibly predict the reproductive potential of gilts, with day 160 circulating AMH levels being a potential predictive indicator.

## 1 Introduction

The reproductive performance of sows is an important factor that affects the economic benefits of farms, so it is crucial to select gilts with high fertility. Currently, gilt selection by traditional body structure, teat number, and vulva size has predicted reproductive performance at some extent; however, it still has limitations in predicting ovarian reserve (1, 2). In addition, reproductive hormone and other indices have been used as predictors, including follicle-stimulating hormone (FSH), inhibin B, estradiol (E_2_), and antral follicle count (AFC). However, these methods cannot accurately predict female reproductive potential owing to hormone causing fluctuating estrus cycles and individual differences (3, 4). Interestingly, circulating AMH levels in women can be detected at any stage and are more stable than other hormones. In clinical practice, AMH is often used in women for assisted reproduction and diagnosis of ovarian dysfunction, with circulating AMH levels predicting the chances of successful ovarian stimulation and subsequent embryonic development (5). In a survey of 769 women in the UK, a customized ovarian stimulation regimen based on individual serum AMH levels resulted in a significant increase in embryo transfer, pregnancy, and live birth rates compared with conventional FSH stimulation therapy (6). Therefore, circulating AMH has emerged as a potential marker for assessing ovarian reserve and antral follicles.

In several species, AMH is only expressed in the granulosa cells of growing follicles (7). During follicular development, AMH is robustly expressed in small antral follicles, with levels decreasing as follicle size increases, and its expression is very low or undetectable in atretic follicles and the corpus luteum (8). However, in pigs, AMH expression has been detected in preovulatory ovarian follicular theca cells and corpora lutea (9). Previous studies have shown that AMH has two major regulatory functions during follicular growth: inhibition of primordial follicle recruitment and reduction of antral follicle sensitivity to FSH (10). *In vivo* studies have shown that AMH-knockout mice exhibit premature ovarian failure due to accelerated follicular growth and more primordial follicles entering the follicular pool (11, 12). *In vitro* studies have shown that exposure of mouse or human ovarian tissue to high levels of AMH results in a significant reduction in the number of growing follicles (13, 14).

To determine whether there is a connection between circulating AMH concentration and the reproductive potential of gilts, the relationship of circulating AMH levels on reproductive performance of 110–160 d-old gilts were examined and evaluated differences in the ovary/uterus of gilts with different AMH levels.

## 2 Materials and methods

### 2.1 Ethics approval and consent to participate

This study was conducted in strict accordance with the Guide for the Care and Use of Agricultural Animals for Agricultural Research and Teaching. The protocol was approved by the Animal Use Committee of Hebei Agricultural University (NO: 2024141).

### 2.2 Animals

The experiment was conducted at the Inner Mongolia Huamu Xingnong Breeding Pig Technology Company. A total of 514 French purebred Large White gilts housed singly in good condition, with healthy limbs and hooves, aged 110–160 days, were selected for the experiment. All gilts selected were given the same husbandry management, including traditional conventional diet and free access to water.

### 2.3 Experimental design

For experiment one, blood samples were collected from gilts (day 110–160) on days 110 (*n* = 66), 120 (*n* = 62), 130 (*n* = 78), 140 (*n* = 112), 150 (*n* = 88), and 160 (*n* = 108) (± 4 days). Circulating AMH levels of gilts were investigated along with the effects of high and low AMH levels on puberty and reproductive performance from across 3 successive parities by measuring circulating AMH levels. Then the AMH levels on different days were grouped according to the quartile method (P_0_ – P_25_ were Low AMH groups, and P_75_ - P_100_ were High AMH groups). Low AMH groups: 110 d < 7.81 ng/mL, 120 d < 9.31 ng/mL, 130 d < 9.83 ng/mL, 140 d < 9.94 ng/mL, 150 d < 11.23 ng/mL, 160 d < 10.86 ng/mL. High AMH groups: 110 d > 10.64 ng/mL, 120 d > 10.99 ng/mL, 130 d > 11.76 ng/mL, 140 d > 12.31 ng/mL, 150 d > 13.55 ng/mL, 160 d > 13.06 ng/mL.

For experiment two, three gilts at age of 160 d with high and low AMH levels (*n* = 3 per treatment) were selected for slaughter. The ovaries were harvested from gilts on the slaughter and transection line in a slaughterhouse. After cutting the animal and highlighting the female genital apparatus, the ovaries were excised with the help of scissors by the same skilled technician. Select gilts with the same or similar physiological statuses for slaughter to obtain ovaries, in order to minimize the impact of individual differences on experimental results. Half of the left ovary was fixed in 4% paraformaldehyde solution for measuring ovarian morphology and the rest were placed in liquid nitrogen. The differences in reproductive organ development and the expression and localization of ovary AMH were compared in the gilts with different circulating AMH levels. The pathways affecting ovarian development in gilts with different AMH levels were analyzed using RNA-Seq.

### 2.4 Estrus detection and artificial insemination

Gilts after age of 160 d were exposed to adult boars twice daily which were rotated every 2 days for detection of onset and duration of their pubertal estrus. For all groups, we defined puberty attainment as the time that gilts first exhibited a standing reflext in response to the back presure test during twice daily (08:00 and 16:00 h) fence contact with two boars at a time. Gilts were then provided their allotted method of boar stimulation for 10-15 min. Mating was carried out on gilts aged 220–240 d following 1–2 obvious estrus cycles, and the same was carried out if the recorded first estrus of gilts occurred after 220 days of age. The first artificial insemination was performed 6 h after standing heat response was detected for gilts, while the second was performed after 24 h. All animals were inseminated with fresh diluted semen (3 × 10^9^ sperm cells, 80% motility, and < 10% malformations), which was also used for the first estrus after age of 220 d. The time of the first mating was recorded as 0 and parity 2 and 3 used the same method as the first breeding. Ultrasound diagnostics (HS-1600 V, Honda, Japan) were performed on sows at day 25 post-mating. For sows in which pregnancy could not be determined, a second examination was performed on day 35 post-mating. Sows were recorded as cull if they were older than 240 d, with no estrus signs, not enter estrus for a long time after weaning, did not conceive after repeated mating, or died due to disease and stress.

### 2.5 Sample collection

In experiment one, blood samples were collected from the anterior vena cava of the gilts into 5-mL vacuum centrifuge tubes which contained a coagulant, and the serum was incubated at 25°C for 30 min to allow clotting. The tubes were then centrifuged at 3,000 r/min for 20 min, and samples were separated and stored at-80°C for storage. In experiment two, after slaughter, uterine and ovarian samples were collected from each gilt, washed with saline, and cut according to the tissue type. The tissue blocks were put into the centrifuge tube and immediately immersed in liquid nitrogen for freezing, marked and wrapped, and immediately stored in the-80°C freezer for use while the other part was stored at room temperature in 4% paraformaldehyde less than 24 h (biotopped, China).

### 2.6 Enzyme linked immunosorbent assay

Circulating AMH levels were quantified using a competitive inhibition ELISA kit (JM-10359P1; Jingmei Biotechnology, China) for porcine AMH. The final concentration of the hormones (ng/mL) was calculated according to the manufacturer’s instructions, and the absorbance was measured at 450 nm using an enzyme marker (SMP500-15370-NSQV, China).

### 2.7 Histomorphology analysis

#### 2.7.1 Hematoxylin and eosin staining

The ovaries and uterus of each gilt were sectioned for histological analysis. Briefly, the tissue samples were washed with PBS three times, and dehydrated in 70, 90% and anhydrous ethanol for 1 h. Next, the tissues were embedded in paraffin wax immersed at 60–70°C for 3 h, fixed at 5 μm in specification and placed in oven at 45°C for drying and were deparaffinized in xylene solution. For hematoxylin and eosin staining, the sections were stained in hematoxylin (30 min), rinsed in running water, and stained in eosin (1 min). H&E staining images were captured with a Nikon D90 digital camera (Nikon, Tokyo, Japan) mounted on a microscope (Olympus, Japan).

#### 2.7.2 Follicle count

The total number of follicles was determined as described by Steel et al. (2). For each gilt, the total follicle count was calculated by multiplying the total number of follicles for each quarter-ovary by a correction factor of 320 (40 × 4 × 2: 40 accounts for counting every 40th section, four accounts for only slicing one-quarter of the ovary, and two accounts for only testing one ovary per gilt).

#### 2.7.3 Endometrial glandular/vascular analysis

Pictures of endometrial areas were saved using the 10x objective using a microscope (Olympus, Japan). Endometrial area (per unit area as mm^2^) was measured individually for each histological field using ImageJ^®^ software (NIH, Bethesda, MD, United States). The total numbers of glands and vessels in the endometrial area were also determined. Glandular density (glands/mm^2^) was determined by dividing the number of glands by the area of the endometrium in the field. The vascular density (vessels/mm^2^) was determined by dividing the number of vessels by the area of the endometrium in the field. Mean glandular/vascular area (μm^2^), as determined by the average area of 50 glands/vessels per photomicrograph, was measured. This experiments was completed by the same technician blinded to the AMH categories for animals.

### 2.8 Immunohistochemical analysis of ovarian tissue

To determine the presence of AMH in porcine ovaries, immunohistochemistry was performed as described by Phoophitphong et al. (15). Briefly, paraffin sections were deparaffinized, treated in a microwave containing ethylenediaminetetraacetic acid (EDTA) (pH 9.0) for 23 min for antigen repair, cooled, and rinsed with PBS (pH 7.4). Sections were incubated in 3% hydrogen peroxide solution at room temperature and protected from light for 25 min to block endogenous peroxidase, and the sectioned tissues were covered with 3% BSA (GC305010, Servicebio, China) dropwise to uniformly cover the tissues and then were closed at room temperature for 30 min. Subsequently, the sections were incubated in wet cassettes with drops of AMH antibody (sc-166752, Santa Cruz, United States), diluted at 1: 100 in PBS, and incubated at 4°C overnight. Next, the sections were incubated with a secondary biotin-labeled goat anti-mouse antibody (GB23301, Servicebio, China) diluted 1:300 in PBS at room temperature for 50 min. Subsequently, DAB color development solution (G1212, Servicebio, China) was added dropwise to the sections, and color development was terminated by rinsing with water. Finally, the sections were re-stained with hematoxylin (G1004, Servicebio, China) for 3 min, placed in 75 and 85% anhydrous ethanol and xylene in sequence for 5 min for dehydration and transparency, respectively, and closed with neutral gum. The results were interpreted by placing under a white light microscope (Olympus, Japan), hematoxylin-stained nuclei were blue and DAB showed positive expression in brownish yellow. Expression level was analyzed using ImageJ installed IHC Toolbox plugin (NIH, Bethesda, MD, United States).

### 2.9 Fluorescent immunohistochemical analysis of uterine tissue

Paraffin sections were deparaffinized, treated in a microwave containing EDTA (pH 9.0) 24 min for antigen repairing. The sectioned tissues were firstly rinsed with 3% BSA (GC305010, Servicebio, China) for 30 min. Subsequently, it was incubated at 4°C overnight in wet cassettes with drops of AMH antibody (sc-166752, Santa Cruz, United States) and Anti-Müllerian hormone type-2 receptor (AMHR2) antibody (ab-060276, Erpan Tech, China), which were diluted at 1: 100 in PBS. Next, the sections were incubated with a secondary biotin-labeled goat anti-mouse antibody (GB23301, Servicebio, China) and goat anti-rabbit secondary antibody (GB25303, Servicebio, China) diluted 1: 300 in PBS at room temperature for 50 min. DAPI staining solution (G1012, Servicebio, China) was added at room temperature for 10 min. Then, fluorescence quenching solution (G1221, Servicebio, China) was added for 5 min, and color development was terminated by rinsing with water. Finally, the sections were sealed with anti-fluorescence quenching sealer (G1401, Servicebio, China). The whole-slide digital images were collected at ×20 magnification with an Aperio Scan Scope Slide Scanner (Leica Biosystems).

### 2.10 RNA extraction, library construction, and sequencing

Total RNA was extracted using a Trizol kit (Invitrogen, Carlsbad, CA, United States) according to the manufacturer’s protocol. RNA quality was assessed using an Agilent 2,100 Bioanalyzer (Agilent Technologies, Palo Alto, CA, United States) and verified via RNase-free agarose gel electrophoresis. After total RNA extraction, the eukaryotic mRNA was enriched with oligo (dT) beads. The enriched mRNA was fragmented into short fragments using fragmentation buffer and reverse transcribed into cDNA using the NEB Next Ultra RNA Library Prep Kit for Illumina (NEB#7530, New England Biolabs, Ipswich, MA, United States). Purified double-stranded cDNA fragments were end-repaired, a base was added, and ligated to Illumina sequencing adapters. The ligation reaction was purified using AMPure XP Beads (1.0X). A polymerase chain reaction (PCR) was then performed. The resulting cDNA library was sequenced using an Illumina Novaseq6000 (Gene Denovo Biotechnology Co., Guangzhou, China). To get high quality clean reads, raw reads containing adapters or low quality bases were further filtered by fastp. RNAs differential expression analysis was performed by DESeq2 software between two different groups. Differentially expressed genes (DEGs) were defined by the criteria of padj <0.05 and |log 2 (fold change) | >1. All DEGs were analyzed for Gene Ontology (GO) enrichment and Kyoto Encyclopedia of the Genome (KEGG) pathway enrichment using the Cluster Profiler R. GO terms and KEGG pathways with *p*-value <0.05 were considered significantly enriched.

### 2.11 Quantitative real-time PCR

Total RNA was extracted using Trizol kit (Invitrogen, Carlsbad, CA, United States) and RNA quality was assessed using NanoDrop 2000 (Thermo, Waltham, MA, United States). Subsequently, cDNA was synthesized by reverse transcription of RNA using Prime Script RT Master Mix (TaKaRa, Japan). QRT-PCR was performed using SYBR fluorescent dye (Biotium, Bay Area, CA, United States) on a QuantStudio 6 Flex Real-Time PCR System (Applied Biosystems). The PCR procedure was as follows: 95°C for 2 min, followed by 40 cycles of 95°C for 5 s and 60°C for 30 s. Relative gene expression level was quantified using 2^−ΔΔCt^ method based on the threshold cycle number. Primers were designed using primer 6.0 (Premier, CA) and the sequences used are listed in Table 1.

**Table 1:** Primers used in this study.

Primers	Primer sequences (5′–3′)	Fragment size (bp)
*GAPDH*	F: GTCGGAGTGAACGGATTTGG	217
	R: TGGAAGATGGTGATGGCCTT	
*AMH*	F: GACATATCAAGCCAACAAC	211
	R: ATCTTAAGCAGAAGCACC	
*AMHR2*	F: ACCACATTGTCCGTTTCATCAC	219
	R: TCAGATCTCGGTGGGCAATAC	
*FOXA2*	F: GAGCGGTGAAGATGGAAG	187
	R: TGAGCGAGATGTACGAGTA	
*VEGF*	F: CTGCTCTACCTCCACCAT	156
	R: TCTTGCCTCGCTCTATCT	
*VEGFR*	F: AAGAGTCACGGAAGAGGAT	221
	R: TAGCAGGAGCCAGAAGAG	
*CD31*	F: GAAGGTGGAGTCGTGAAG	204
	R: CGTGTAGTTGCTGTTGTG	
*AKR1C3*	F: ACGACGGTCACTTCATTCCT	241
	R: TTCTCTCTTCACGGTGCCAT	
*SRD5A2*	F: CTGCATGGGAAATGGACTCC	206
	R: AACGTACGTGAACAAGCCAC	
*PLA2G4E*	F: CCCCTTGATTGCCTCTCAGA	165
	R: CAACACCTGCTTCAATGCCT	
*FOS*	F: CTTTGCAGACTGAGATCGCC	196
	R: CTGCTGACGTTCTTGACTGG	
*FGF10*	F: CGTCCTTCTCCTCTCCTT	203
	R: TCCTCTATCCTCTCCTTCAG	
*GRIA1*	F: TTGCTTTGTCCCAACTCACG	214
	R: TGTGTCAACGGGAAAGCTTG	

### 2.12 Western blot

Tissues were placed in centrifuge tubes, ground with liquid nitrogen, and lysed with lysis buffer containing protease inhibitors (Biotopped, China). After centrifuging at 12,000 rpm for 20 min at 4°C, the supernatant was aspirated and placed in 95°C metal bath for 10 min.BCA (Biomed, China) was used to determine the concentration of sample protein. After SDS-PAGE (120 V, 0.04 mA, 40 min) electrophoresis, proteins were transferred to NC membranes (Biotopped, China). and were incubated with AMH antibody (Sc-166752; Santa Cruz, United States) at 4°C overnight. Horseradish peroxidase-conjugated goat anti-mouse IgG (bs-0296G-HRP; Bioss, China) was diluted 1:10,000. then the membranes were incubated with secondary antibody at room temperature for 50 min, and washed with TBST three times. Finally, luminescent chromogenic solutions were configured using Beyo ECL Plus (P0018S, Beyotime, China) and images were acquired on a chemiluminescent imaging analyzer (Tanon, China).

### 2.13 Statistical analysis

The obtained data were statistically analyzed by SPSS version 21.0 (IBM Corporation, Armonk, NY, United States). The normal distribution and homogeneity of variance were tested. The Student’s independent t test was employed to reveal the significance between the High-AMH and Low-AMH groups. The Student’s independent chi-square test was employed to reveal the significance AMH Level among mutiple groups. *p*-values <0.05 and < 0.01 were considered significant differences and extremely significant differences, respectively. The data have been presented as mean ± SD. The relationship between AMH levels at 110–160 days of age and puberty was compared using correlations (Graphpad Software, SD, CA, United States).

## 3 Results

### 3.1 Anti-Müllerian hormone levels in gilts from 110 to 160 d of age and its correlation to puberty

**Figure 1:**
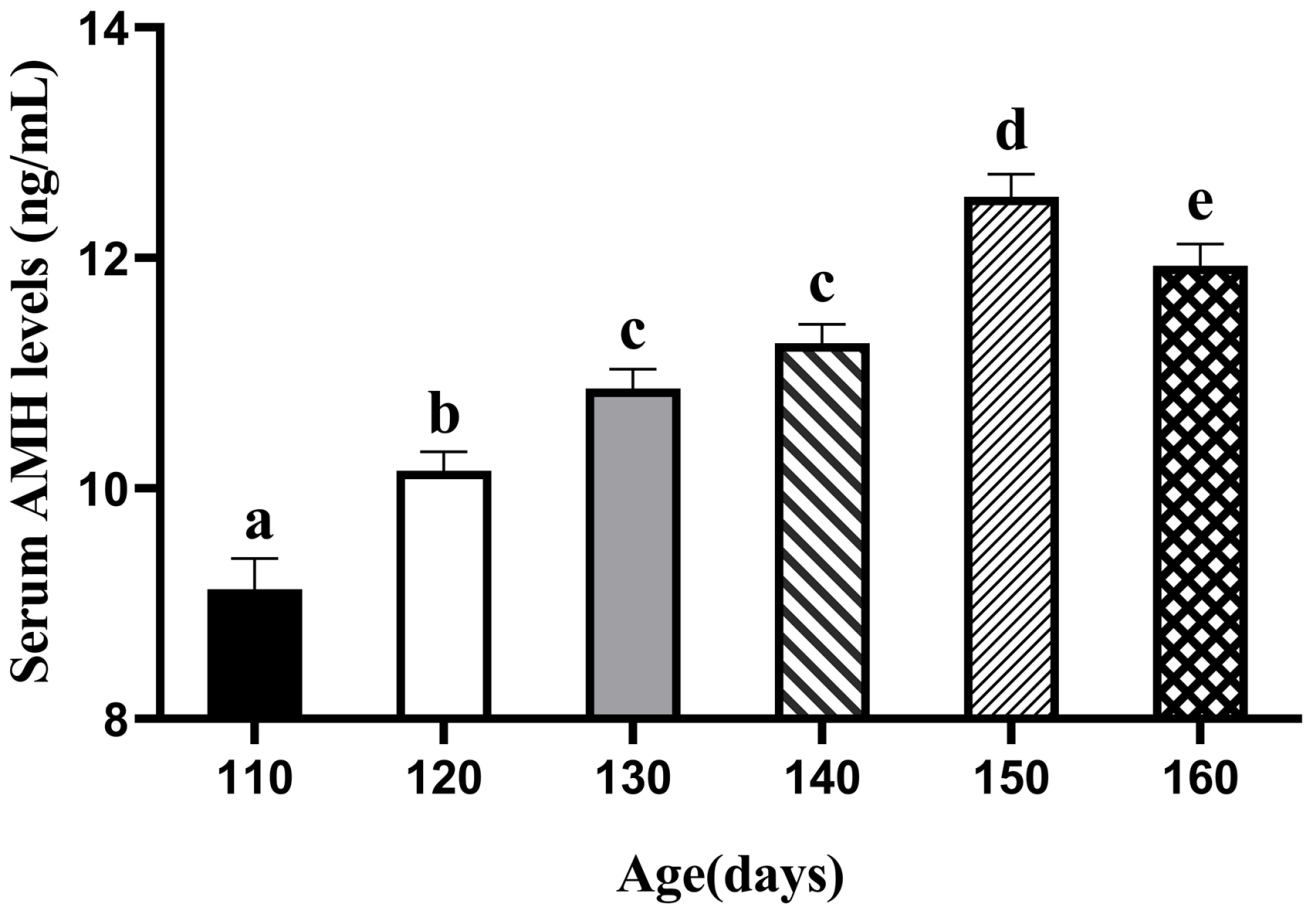
Trends in AMH levels in gilts from 110 to 160 days of age. (At 110 days *N* = 66; At 120 days *N* = 62; At 130 days *N* = 78; At 140 days *N* = 112; At 150 days *N* = 88; At 160 days *N* = 108). a,b,c,d,e Values within rows with different superscripts differ (*p* < 0.05).

**Figure 2:**
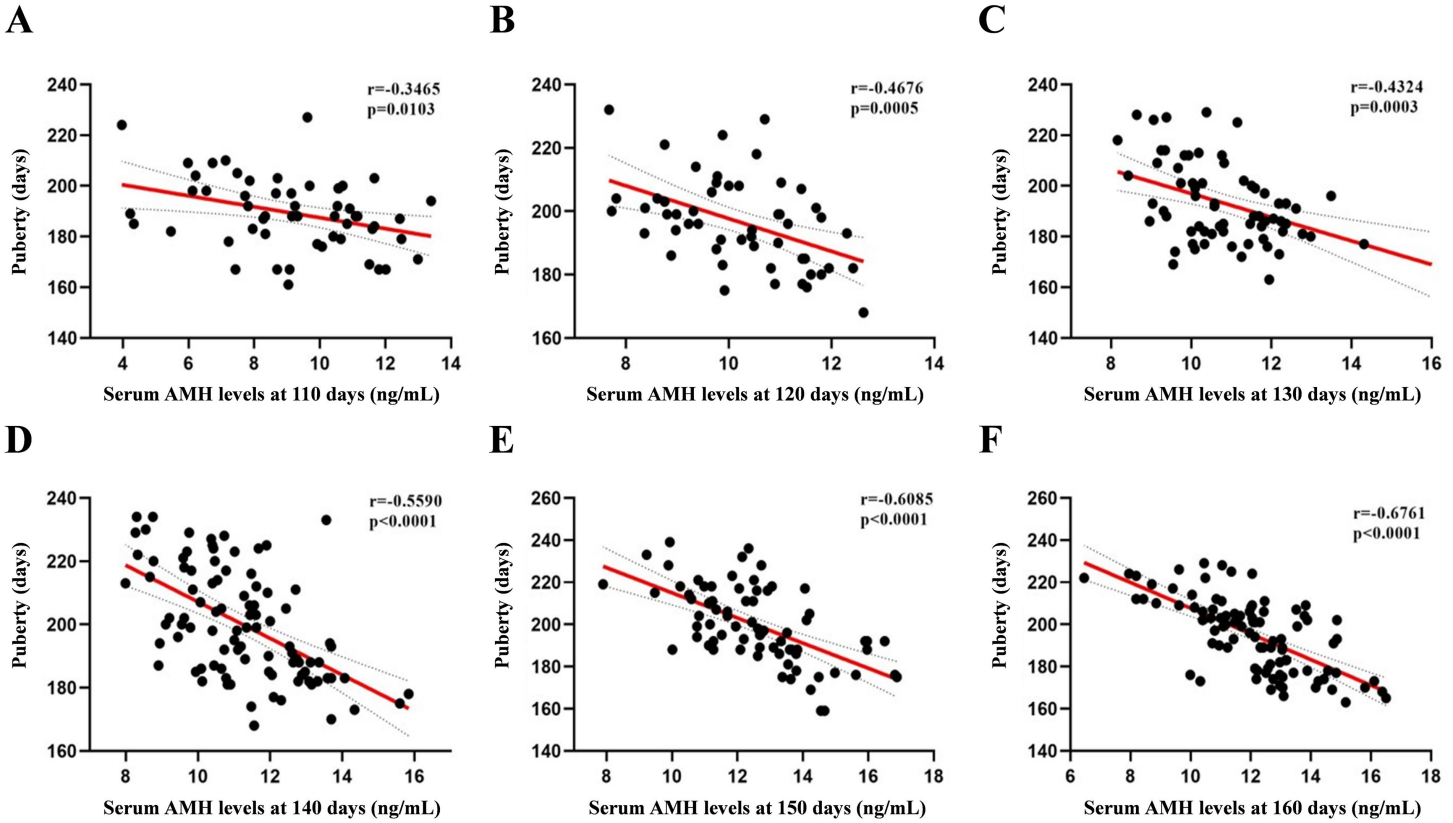
Pearson’s correlation coefficients between AMH levels from 110 to 160 days of age and puberty. **(A)** At 110 days *N* = 54, **(B)** at 120 days *N* = 52, **(C)** at 130 days *N* = 67; **(D)** at 140 days *N* = 94; **(E)** at 150 days *N* = 75; **(F)** at 160 days *N* = 95.

We first attempted to determine the circulating AMH levels in gilts from day 110 to 160, as shown in Figure 1. AMH levels increased from days 110 to 150 and peaked at day 150 (12.53 ng/mL). The relationship between circulating AMH levels and puberty in gilts was investigated using Pearson’s correlation analysis, and a significant negative correlation was found between gilts on days 110 and 160, as shown in Figure 2 (*p* < 0.05). These results suggest that gilts with high circulating AMH levels experienced early puberty.

### 3.2 Effects of circulating AMH levels on the reproductive performance of sows from across 3 successive parities

As shown in Tables 2–4, in the first parity, the total birth rate/litter was significantly higher in gilts with high-AMH levels at day 130, 140, and 160 (*p* < 0.05), and the liveborn rate was also significantly higher in gilts with high-AMH levels than gilts with low-AMH levels at day 160 of age (*p* < 0.01). In the second parity, the total birth rate in the litter was significantly higher in gilts with high-AMH levels than gilts with low-AMH levels at day 120, 140, 150, and 160 of age (*p* < 0.05), and the liveborn rate was also significantly higher in gilts with high-AMH levels than low-AMH ones gilts with low-AMH levels at day 140, 150, and 160 of age (*p* < 0.05). In addition, for third parity, total births were significantly higher in the gilts with high-AMH levels than gilts with low-AMH levels at day 110, 140, and 150 of age (*p* < 0.05).

**Table 2:** Effect of different AMH levels on the reproductive performance of sows with 1 litter.

Group		N	AMH Levels/ng/mL	Total born/litter	Liveborn /litter	Stillborn/litter	Average litter weight/kg
110	High-AMH	16	11.68 ± 0.82^A^	15.44 ± 2.68	13.25 ± 1.29^a^	2.00 ± 1.79	1.23 ± 0.09
	Low-AMH	15	6.29 ± 1.29^B^	13.87 ± 2.53	11.73 ± 1.87^b^	2.33 ± 1.99	1.27 ± 0.11
120	High-AMH	16	11.66 ± 0.47^A^	15.81 ± 2.61	13.75 ± 2.02	2.06 ± 1.84	1.32 ± 0.16
	Low-AMH	14	8.59 ± 0.53^B^	14.36 ± 3.15	12.57 ± 3.27	1.79 ± 1.85	1.30 ± 0.07
130	High-AMH	19	12.61 ± 1.16^A^	16.05 ± 3.42^a^	13.26 ± 2.18	2.68 ± 3.02^a^	1.24 ± 0.10
	Low-AMH	17	9.21 ± 0.46^B^	13.06 ± 3.45^b^	11.82 ± 3.94	1.12 ± 1.58^b^	1.28 ± 0.16
140	High-AMH	26	13.35 ± 0.87^A^	16.00 ± 2.98^A^	12.65 ± 2.46	2.46 ± 2.40	1.29 ± 0.18
	Low-AMH	23	9.15 ± 0.61^B^	13.22 ± 3.85^B^	11.22 ± 3.64	1.65 ± 1.72	1.27 ± 0.14
150	High-AMH	22	14.76 ± 1.12^A^	15.05 ± 3.36	12.68 ± 2.06	1.82 ± 1.99	1.44 ± 0.20
	Low-AMH	19	10.41 ± 0.85^B^	13.00 ± 3.99	11.21 ± 4.04	1.63 ± 1.64	1.41 ± 0.22
160	High-AMH	26	14.34 ± 1.03^A^	15.61 ± 3.64^A^	13.08 ± 3.22^A^	1.69 ± 1.38	1.50 ± 0.20
	Low-AMH	23	9.63 ± 1.17^B^	11.78 ± 2.95^B^	10.22 ± 3.22^B^	1.30 ± 1.40	1.56 ± 0.28

Data are presented as mean ± SEM. ^a,b^ Values notations within age group with different superscripts differ (*P* < 0.05). ^A,B^ Values notations within age group with different superscripts differ (*P* < 0.01).

**Table 3:** Effect of different AMH levels on the reproductive performance of sows with 2 litter.

Group		N	AMH Levels/ng/mL	Total born/litter	Liveborn /litter	Stillborn/litter	Average litter weight/kg
110	High-AMH	14	11.66 ± 0.83^A^	16.86 ± 2.93	14.00 ± 2.29	2.71 ± 2.16	1.32 ± 0.16
	Low-AMH	13	6.37 ± 1.24^B^	14.85 ± 4.16	12.15 ± 2.91	2.69 ± 1.89	1.32 ± 0.15
120	High-AMH	13	11.66 ± 0.41^A^	17.46 ± 3.84^a^	14.23 ± 3.11	3.23 ± 2.59	1.43 ± 0.14
	Low-AMH	12	8.59 ± 0.57^B^	14.08 ± 4.14^b^	11.67 ± 3.31	2.00 ± 1.48	1.47 ± 0.22
130	High-AMH	17	12.65 ± 1.21^A^	16.94 ± 4.34	14.24 ± 3.70	2.18 ± 2.13	1.44 ± 0.30
	Low-AMH	12	9.13 ± 0.50^B^	14.67 ± 4.62	11.50 ± 4.17	2.58 ± 1.73	1.55 ± 0.25
140	High-AMH	24	13.41 ± 0.88^A^	16.46 ± 3.27^a^	13.67 ± 2.26^a^	2.67 ± 2.14	1.40 ± 0.19
	Low-AMH	20	9.24 ± 0.57^B^	13.40 ± 4.38^b^	11.25 ± 3.91^b^	1.75 ± 1.71	1.41 ± 0.20
150	High-AMH	18	14.79 ± 1.18^A^	16.17 ± 3.01^a^	14.06 ± 3.06^a^	2.11 ± 1.84	1.27 ± 0.10
	Low-AMH	13	10.34 ± 0.94^B^	13.08 ± 4.52^b^	11.15 ± 3.53^b^	1.92 ± 1.85	1.33 ± 0.20
160	High-AMH	23	14.29 ± 0.99^A^	16.56 ± 4.76^a^	13.96 ± 3.94^A^	2.52 ± 3.03	1.22 ± 0.14
	Low-AMH	17	9.38 ± 1.27^B^	12.29 ± 5.50^b^	10.00 ± 5.05^B^	2.06 ± 1.78	1.36 ± 0.44

Data are presented as mean ± SEM. ^a,b^ Values notations within age group with different superscripts differ (*P* < 0.05). ^A,B^ Values notations within age group with different superscripts differ (*P* < 0.01).

**Table 4:** Effect of different AMH levels on the reproductive performance of sows with 3 litter.

Group		N	AMH Levels/ng/ml	Total born/litter	Liveborn /litter	Stillborn/litter	Average litter weight/kg
110	High-AMH	7	12.10 ± 0.88^A^	17.29 ± 2.43^a^	15.00 ± 2.65	2.14 ± 1.35	1.29 ± 0.10
	Low-AMH	8	6.60 ± 1.16^B^	15.00 ± 1.51^b^	13.63 ± 1.77	1.25 ± 1.04	1.30 ± 0.08
120	High-AMH	9	11.53 ± 0.32^A^	17.78 ± 3.63	14.89 ± 2.52	2.56 ± 2.74	1.26 ± 0.12
	Low-AMH	7	8.77 ± 0.57^B^	15.86 ± 2.85	13.43 ± 2.44	2.29 ± 1.11	1.27 ± 0.06
130	High-AMH	14	12.34 ± 0.66^A^	17.86 ± 3.57	14.93 ± 3.27	2.57 ± 2.17	1.28 ± 0.09
	Low-AMH	8	9.16 ± 0.35^B^	15.43 ± 2.51	13.57 ± 2.37	1.71 ± 0.76	1.28 ± 0.08
140	High-AMH	17	13.47 ± 1.01^A^	18.12 ± 3.16^a^	15.29 ± 2.49	2.76 ± 2.59	1.35 ± 0.11
	Low-AMH	14	9.48 ± 0.41^B^	15.43 ± 2.34^b^	13.93 ± 2.13	1.64 ± 1.65	1.42 ± 0.10
150	High-AMH	12	14.86 ± 1.38^A^	17.75 ± 2.38^a^	14.17 ± 2.12	3.17 ± 1.53	1.33 ± 0.11
	Low-AMH	9	10.36 ± 0.59^B^	14.56 ± 3.81^b^	12.11 ± 3.10	1.89 ± 1.36	1.36 ± 0.13
160	High-AMH	19	14.34 ± 1.04^A^	16.05 ± 3.44	13.00 ± 4.16	2.84 ± 4.23	1.35 ± 0.16
	Low-AMH	14	9.52 ± 1.33^B^	14.00 ± 4.07	11.14 ± 4.99	2.57 ± 2.50	1.32 ± 0.16

Data are presented as mean ± SEM. ^a,b^ Values notations within age group with different superscripts differ (*P* < 0.05). ^A,B^ Values notations within age group with different superscripts differ (*P* < 0.01).

To investigate the effect of circulating AMH levels on the productive life span of gilts, as shown in Table 5, gilts in the High–AMH group had a higher retention of across 3 successive parities, but the retention of the third parity sows was not different in the High-AMH group compared with the Low-AMH group (*p* > 0.05).

**Table 5:** Effect of different AMH levels on retention in sows.

Groups		1 Parity	*P*-value	2 Parity	*P*-value	3 Parity	*P-*value
110	High-AMH	(16/17) 94.12%	0.559	(14/17) 82.35%	0.683	(7/17) 41.18%	0.739
	Low-AMH	(15/17) 88.23%		(13/17) 76.47%		(8/17) 47.06%	
120	High-AMH	(16/16) 100%	0.154	(13/16) 81.25%	0.681	(9/16) 56.25%	0.495
	Low-AMH	(14/16) 87.50%		(12/16) 75.00%		(7/16) 43.75%	
130	High-AMH	(19/20) 95.00%	0.305	(17/20) 85.00%	0.080	(14/20) 70.00%	0.059
	Low-AMH	(17/20) 85.00%		(12/20) 60.00%		(8/20) 40.00%	
140	High-AMH	(26/28) 92.86%	0.234	(24/28) 85.71%	0.200	(17/28) 60.71%	0.429
	Low-AMH	(23/28) 82.14%		(20/28) 71.43%		(14/28) 50.00%	
150	High-AMH	(22/22) 100%	0.083	(18/22) 81.82%	0.103	(12/22) 54.55%	0.377
	Low-AMH	(19/22) 86.36%		(13/22) 59.09%		(9/22) 40.91%	
160	High-AMH	(26/27) 96.30%	0.167	(23/27) 85.19%	0.065	(19/27) 70.37%	0.169
	Low-AMH	(23/27) 85.19%		(17/27) 62.96%		(14/27) 51.85%	

Retention rate of sows in the Nth lactation = number of sows delivered in the Nth lactation/total number of sows in the subgroup × 100%.

### 3.3 Anti-Müllerian hormone histological differences in uterus

**Figure 3:**
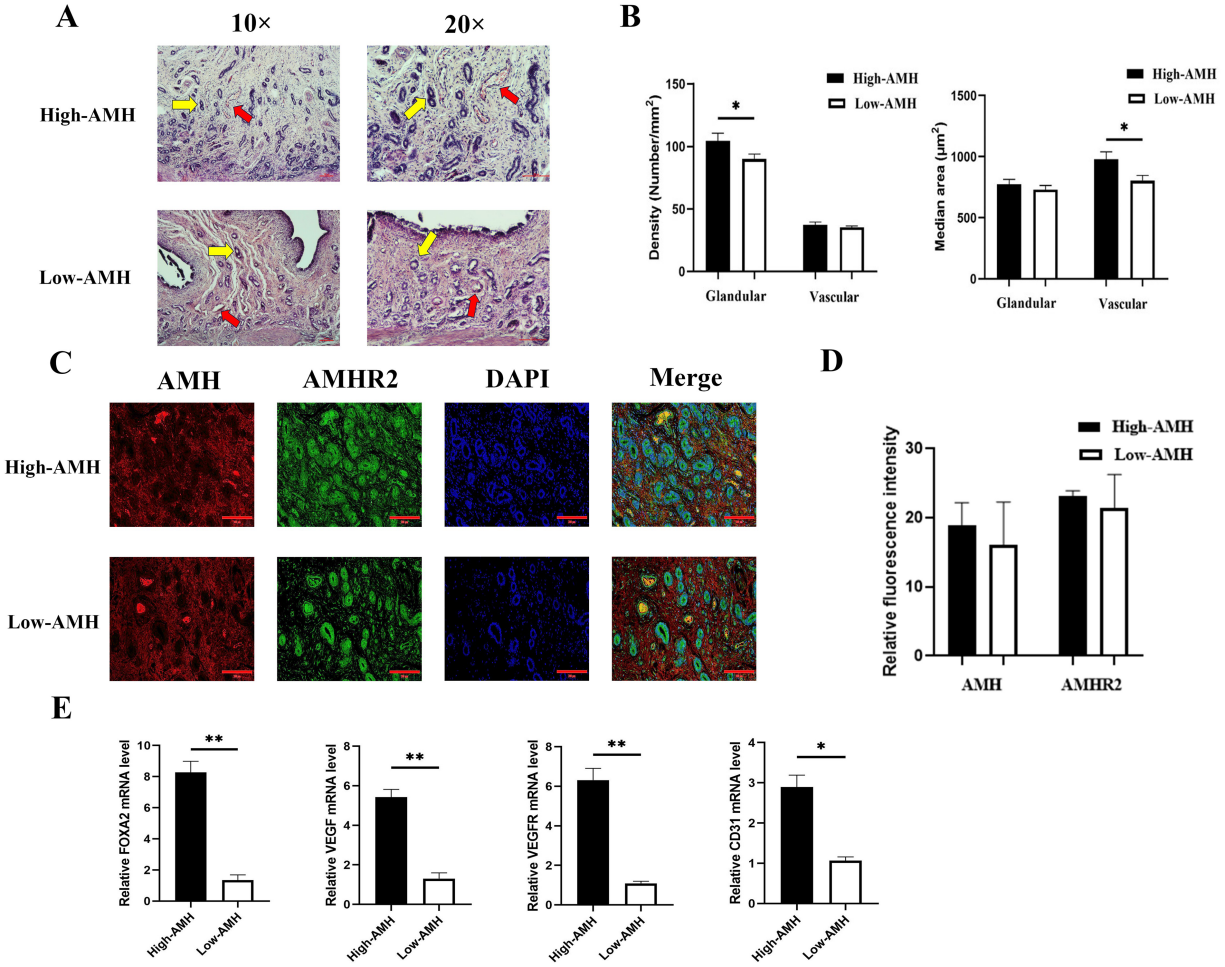
High levels of AMH promote the development of uterine glands and blood vessels in gilts. **(A)** HE staining images of uterine tissue sections from gilts with different AMH levels. Yellow arrows indicate endometrial glandular, red arrows indicate uterine vascular. 10× and 20 × Bar = 100 μm. **(B)** Evaluation of endometrial glands and blood vessels in the endometrium of gilts with different AMH levels. GD, glandular density (glands/mm^2^); MGA, median glandular area (μm^2^/gland); VD, vascular density (vessels/mm^2^); MVA, median vascular area (μm^2^/gland). **(C)** The fluorescence images of uterine staining. Bar = 200 μm. **(D)** The relative fluorescence intensity of AMH and AMHR2 staining. **(E)** The mRNA relative expression level of uterine gland development and angiogenesis genes in gilts with different AMH levels. * Mean significant difference between groups with *p* < 0.05; ** mean significant difference between groups with *p* < 0.01.

To investigate uterine morphology of gilts with different AMH levels, staining and the expression of relevant genes were analyzed. High AMH were found correlated with greater uterine glandular density (104.34 ± 6.12 vs. 89.95 ± 3.89) and median vascular area (979.00 ± 429.54 vs. 802.12 ± 316.74) in gilts (*p* < 0.05, Figures 3A,B). Immunofluorescence results showed that AMH was expressed only in vascular tissue, whereas AMHR2 whose full name is anti-Mullerian hormone receptor, type II and AMH exerts its role mainly through the AMHR2 was expressed in vascular tissue, glandular cells, and stromal cells, but there was no significant difference in fluorescence intensity levels of AMH and anti-Mullerian hormone receptor type II (AMHR2) between the High-AMH and the Low-AMH group (*p* > 0.05) (Figures 3C,D). In addition, qRT-PCR results showed that the relative expression levels of *FOXA*2 (uterine adhesion/development), *VEGF*, *VEGFR* (angiogenesis), and *CD31* (immune response) were significantly increased in high-AMH gilts (*p* < 0.05) (Figure 3E). Taken together, these findings suggest that high AMH levels were beneficial for uterine development.

### 3.4 Anti-Müllerian hormone histological differences in ovary

**Figure 4:**
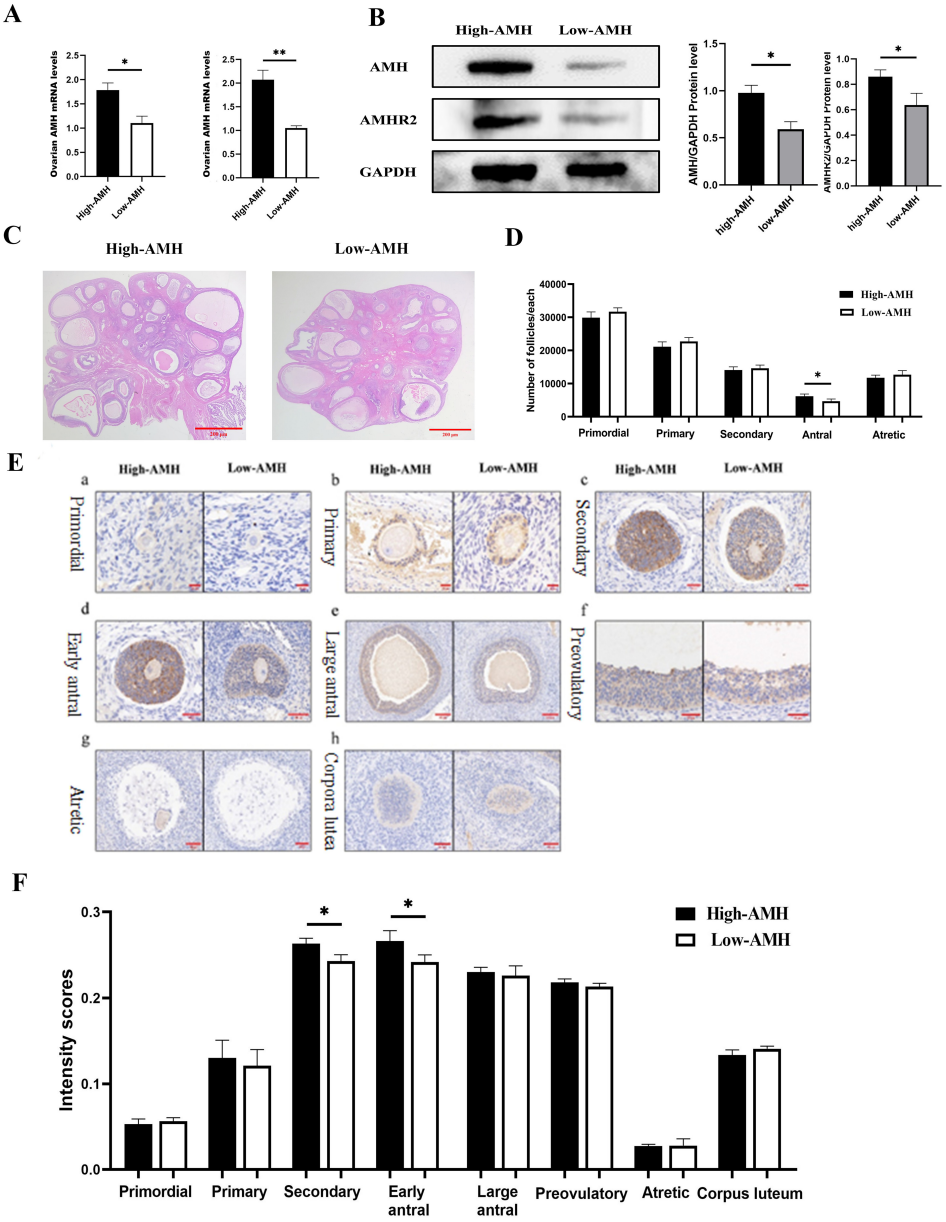
High levels of AMH are associated with the number of antral follicles in gilts. **(A)** Detection of mRNA level of Ovarian AMH and AMHR2 genes by qRT-PCR. **(B)** Detection of Ovarian AMH and AMHR2 protein level and Quantitative level. **(C)** HE staining images of ovarian tissue sections from gilts with different AMH levels. Bar = 100 μm. **(D)** Statistics of follicle counts in gilts with different AMH levels. **(E)** Intensity of AMH immunohistochemistry at different Stages of follicular from gilts with different AMH levels. **(a)**. Primordial **(b)**. Primary **(c)**. Secondary **(d)**. Early antral **(e)**. Large antral **(f)**. Preovulatory **(g)**. Atretic **(h)**. Corpora lutea. Bar = 20, 50, 100, 200 μm. **(F)** Intensity scores (mean ± SD) of AMH immunolocalization at different Stages of follicular from gilts with different AMH levels. * Mean significant difference between groups with *p* < 0.05; ** mean significant difference between groups with *p* < 0.01.

Circulating AMH levels reflect the mRNA and protein levels of ovarian AMH and Anti-Müllerian hormone type-2 receptor (AMHR2), which were significantly higher in the High-AMH than in the Low-AMH group (*p* < 0.05) (Figures 4A,B). The H&E staining results of ovaries with different AMH levels are shown in Figure 4C. The number of antral follicles was significantly higher in the High-AMH group than in the Low-AMH one (*p* < 0.05) (Figure 4D).

To further investigate the localization and expression of AMH in the ovaries, immunohistochemistry were performed. AMH was found mainly expressed in the granulosa cells and showed an increase and then a decrease with follicular development. AMH had the highest immunological intensity in early antral follicles, whereas it was almost not expressed in atretic follicles. AMH was expressed in the corpus luteum of gilts. Furthermore, comparing the different AMH levels in gilts, the High-AMH group had a significantly higher expression level in secondary and early antral follicles than the Low-AMH group (*p* < 0.05) (Figures 4E,F).

### 3.5 Determining the transcriptional landscape of gilt ovaries with different AMH levels

**Figure 5:**
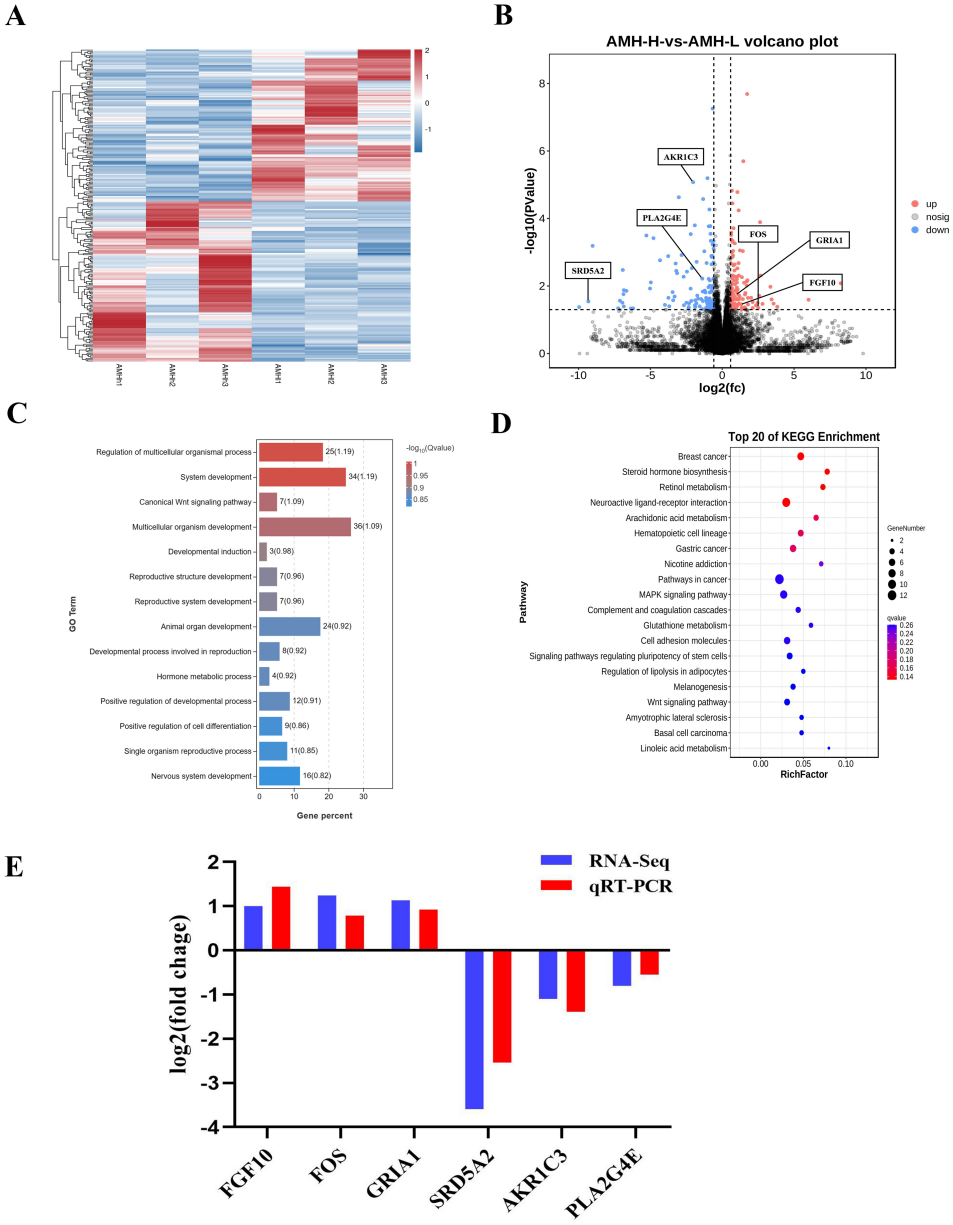
Identification of the transcriptional landscapes of Ovaries of gilts with different AMH levels. **(A)** Heatmap of differential expressed genes in the High-AMH and Low-AMH ovarian. H1, H2, and H3 represent three replicates of the High-AMH, and I1, I2, I3 represent three replicates of the Low-AMH. **(B)** Volcano plot showing transcriptomic landscapes in High-AMH and Low-AMH. **(C)** GO enrichment analysis showing 14 pathways of the DEGs. **(D)** KEGG pathway analysis showing 11 pathways of DEGs involved in Ovaries of gilts with different AMH levels. **(E)** The mRNA expression levels of six genes (*SRD5A2*, *AKR1C3*, *PLA2G4E*, *FGF10*, *FOS*, *GRIA1*) in ovarian from High-AMH and Low-AMH. * Mean significant difference between groups with *p* < 0.05; ** mean significant difference between groups with *p* < 0.01.

RNA-seq analysis of ovaries with high AMH and low AMH levels were further performed. Cluster analysis revealed that the transcripts of the High-AMH group were clearly separated from those of the Low-AMH group (Figure 5A). A total of 241 genes were identified as significantly differentially expressed in the High-AMH group, with 117 up-regulated and 124 down-regulated (Figure 5B).

Functional enrichment (GO) analysis was performed to further investigate the signaling pathways affected by different circulating AMH levels. The results showed that hormone metabolic processes, reproductive structure, and system development were significantly enriched (Figure 5C). KEGG analysis of the DEGs revealed increased expression of genes related to steroid hormone biosynthesis, arachidonic acid metabolism, and the gonadotropin-releasing hormone (GnRH) signaling pathway in the High-AMH group (Figure 5D).

QRT-PCR analysis was performed to verify the changes in gene expression identified using RNA-Seq. Six genes were selected for verification based on their functions in reproductive system development and steroid hormone biosynthesis. The results revealed that the expression levels of six genes including down-regulated (*SRD5A2*, *AKR1C3*, *PLA2G4E*), and up-regulated (*FGF10*, *FOS*, and *GRIA1*) were consistent with the RNA-seq results (Figure 5E).

## 4 Discussion

Gilts with early puberty usually have greater reproductive potential, primarily because of the reduced number of non-productive days and increased lifetime litter size (16). Previous reports have indicated that higher prepubertal AMH levels are associated with a younger age at puberty in pigs (17). In the present study, a significant negative correlation was found between circulating AMH levels and age at puberty in gilts. Furthermore, circulating AMH levels were found positively correlated with the number of antral follicles, suggesting that prepubertal circulating AMH levels reflect the size of antral follicular pools in gilts (18). A study in pigs reported that lower circulating AMH levels were associated with fewer antral follicles (19). Studies on cattle have reached similar conclusions, suggesting that elevated circulating AMH levels may be due to an increased number of ovarian antral follicles (20, 21). Therefore, circulating AMH have been shown to be a good indicator of ovarian reserve and AFC (22).

Interestingly, high-AMH gilts had greater reproductive performance, with a significantly greater total and live births per litter, whereas low-AMH gilts were at risk of early culling, suggesting that the reproductive potential of females is related to the number of antral follicles. Low AMH levels may also increase the risk of ovarian failure (23). Lahoz et al. found that ewes with high AMH levels were 34.8% more fertile at first mating than ewes with low AMH levels (24). It has also been reported that circulating AMH levels are closely related to predicting the ovarian response in dairy cows and that cows with high AMH levels have an increased number of ovulations and transferable embryos after FSH stimulation (25, 26). In addition, circulating AMH levels in dairy cows prior to the first mating were positively correlated with pregnancy rate and number of parities (27). These results suggest that high AMH levels are critical for predicting the reproductive potential of gilts, cattle, and sheep.

To determine the localization and expression of AMH in gilts with different circulating AMH levels, an ovarian immunohistochemical analysis of AMH were performed. The results showed that AMH was mainly expressed in granulosa cells and remained highly expressed in preovulatory follicles and the corpus luteum, with the highest expression in early antral follicles and virtually no expression in atretic follicles. This expression pattern is not similar to that in other species, in which previous publications have found that 5–8 mm follicles contribute 60% of the circulating AMH in humans, which decreases dramatically when the follicle diameter exceeds 8 mm, and AMH expression is virtually undetectable in the granulosa cells of preovulatory follicles (28). Currently, pigs are the only species in which AMH expression remains in the follicle and corpus luteum prior to ovulation, which is physiologically important for maintaining sow fertility. It has been found that the persistence of AMH in the corpus luteum after ovulation may be beneficial in decreasing the response of small antral follicles to FSH, which in turn prevents premature follicular recruitment and atresia from occurring (9). Interestingly, the expression level found in the secondary and early antral follicles was significantly higher in gilts with high AMH levels than in those with low AMH levels, which may be a major reason for the elevated circulating AMH levels. This is consistent with the results of Tienthai et al., who reported that the lower intensity of AMH immunostaining in the growing follicles of the ovaries of non-estrus gilts may be related to ovarian physiological dysfunction due to insufficient circulating AMH levels (29).

GnRH is essential for the onset of puberty and reproductive development, and a growing number of studies have revealed the regulatory function of AMH (30, 31). Anti-Müllerian hormone binds to a specific receptor AMHR2 (anti-Mullerian hormone receptor type II) that regulates the expression of its target genes (32). Here, RNA-seq analysis of ovaries were performed with different AMH levels and found that high levels of AMH activation may be related to receptors in the hypothalamus that promote the synthesis of steroid hormones, thereby affecting the development of reproductive organs and the occurrence of puberty. Malone et al. used whole-exome sequencing to determine whether AMH is expressed in mouse and human fetal migratory GnRH neurons and showed that deficient AMH signaling leads to pubertal deficits and infertility (33). Anti-Müllerian hormone has also been shown to increase GnRH-dependent luteinizing hormone pulsation and secretion, supporting the central effect of AMH on GnRH neurons (34). However, in women, AMHR2 is highly expressed mainly in the gonads and nerve centers, and the functions of AMH and AMHR2 have not been extensively studied, except for their major role in the ovary. Therefore, the mechanism underlying the effects of high circulating AMH levels on puberty and fertility in gilts requires further exploration.

## 5 Conclusion

In conclusion, our findings indicate that circulating AMH levels can possibly predict the reproductive potential of gilts, high-AMH gilts exhibit better reproductive performance than low-AMH ones, and the day 160 circulating AMH levels is a potential predictive indicator.

## Data Availability

All data supporting our findings are included in the manuscript. The datasets presented in this study can be found in online repositories. The names of the repository/repositories and accession number(s) can be found below: NCBI SRA (BioProject lD. PRJNA1215573).

## Supplementary material

Supplementary Figure 1 Western blot of GAPDH gene (note High AMH group on the left; Low AMH group on the right).

Supplementary Figure 2 Western blot of AMH gene (note High AMH group on the left; Low AMH group on the right).

Supplementary Figure 3 Western blot of AMHR2 gene (note High AMH group on the left; Low AMH group on the right).

Supplementary Figure 4 H&E Staining Diagram of Ovarian Tissue in High-AMH Group.

Supplementary Figure 5 H&E Staining Diagram of Ovarian Tissue in Low-AMH Group.

Supplementary Figure 6 H&E Staining Diagram of Uterine Tissue in High-AMH Group.

Supplementary Figure 7 H&E Staining Diagram of Uterine Tissue in Low-AMH Group.
