# Supplementary figures and images for: Effect of circulating anti-Mullerian hormone on the reproductive potential of gilts
Source: Front Vet Sci. 2025 Feb 27;12:1454343. doi: 10.3389/fvets.2025.1454343 (PMC11904792; doi:10.3389/fvets.2025.1454343)

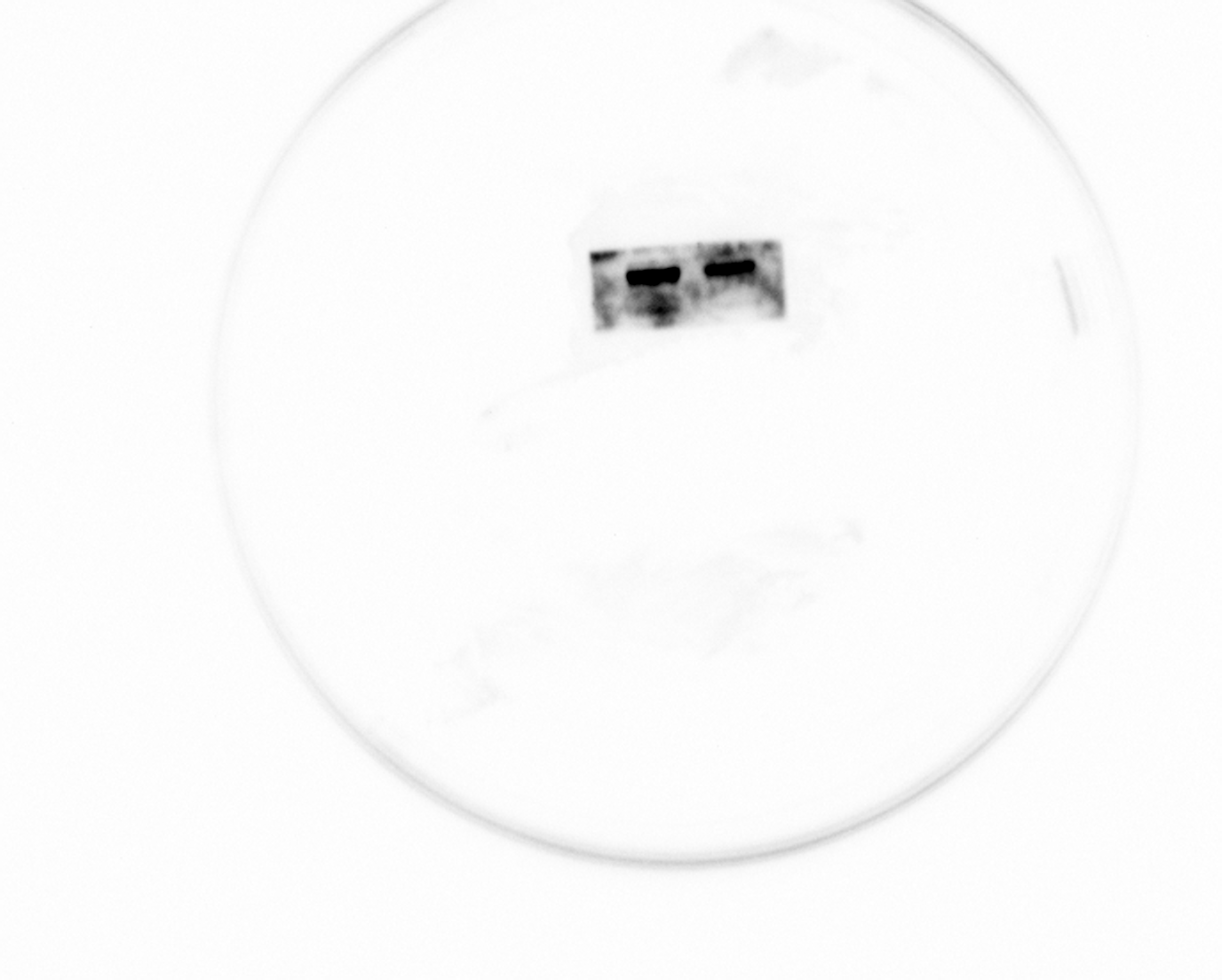

Supplement: Supplementary Figure 1 — Western blot of GAPDH gene (note High AMH group on the left; Low AMH group on the right). [file Image_1.tif]

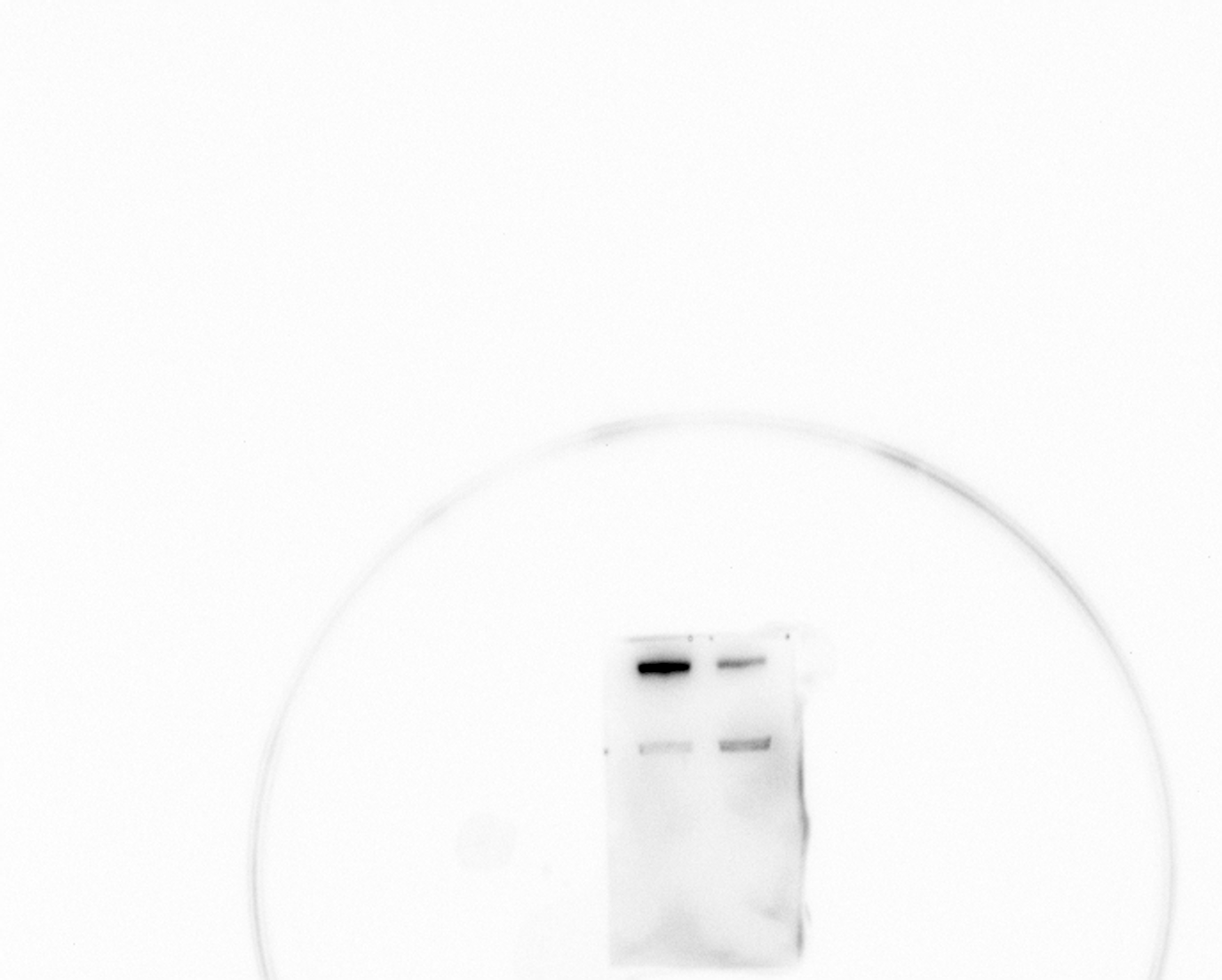

Supplement: Supplementary Figure 2 — Western blot of AMH gene (note High AMH group on the left; Low AMH group on the right). [file Image_2.tif]

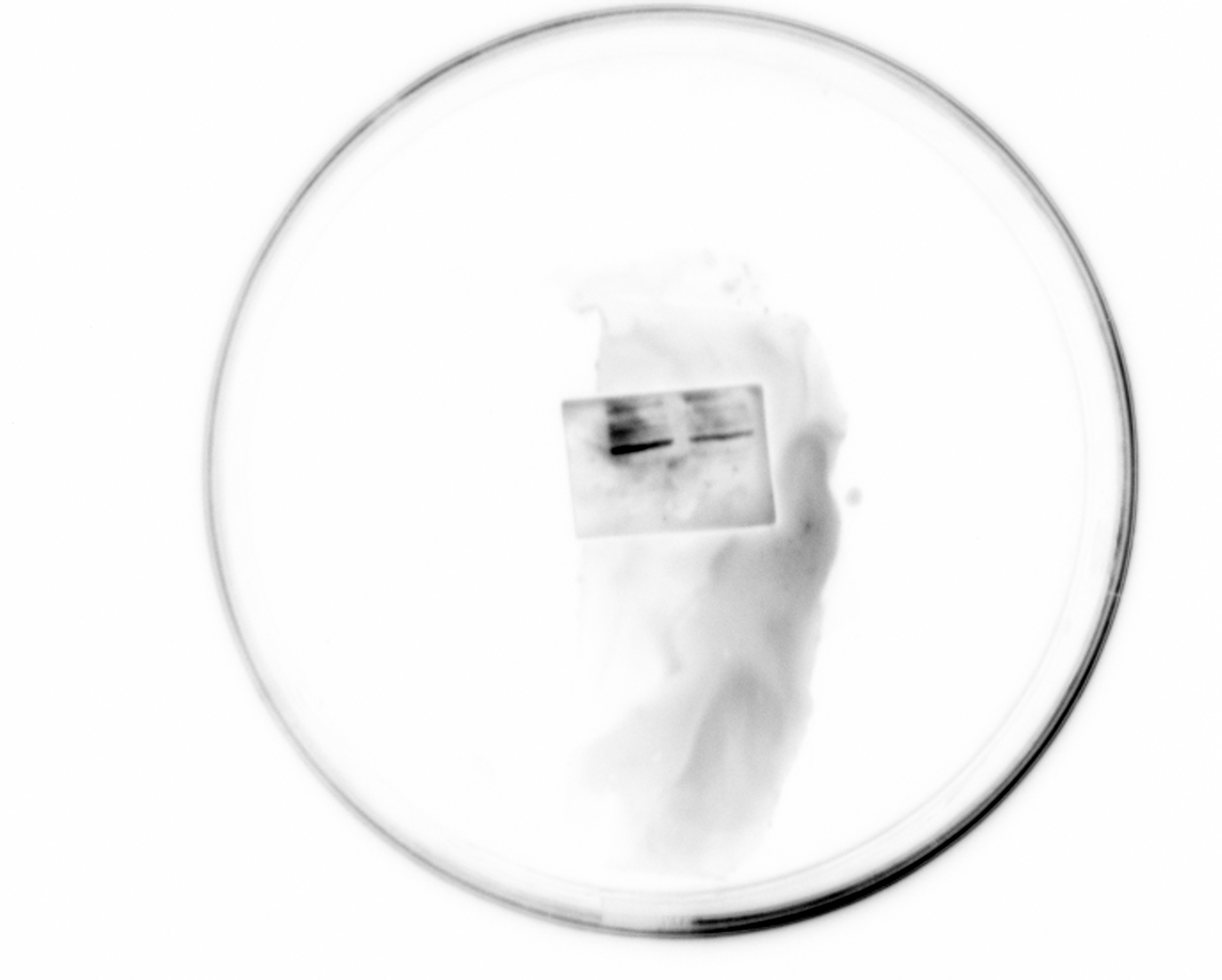

Supplement: Supplementary Figure 3 — Western blot of AMHR2 gene (note High AMH group on the left; Low AMH group on the right). [file Image_3.tif]

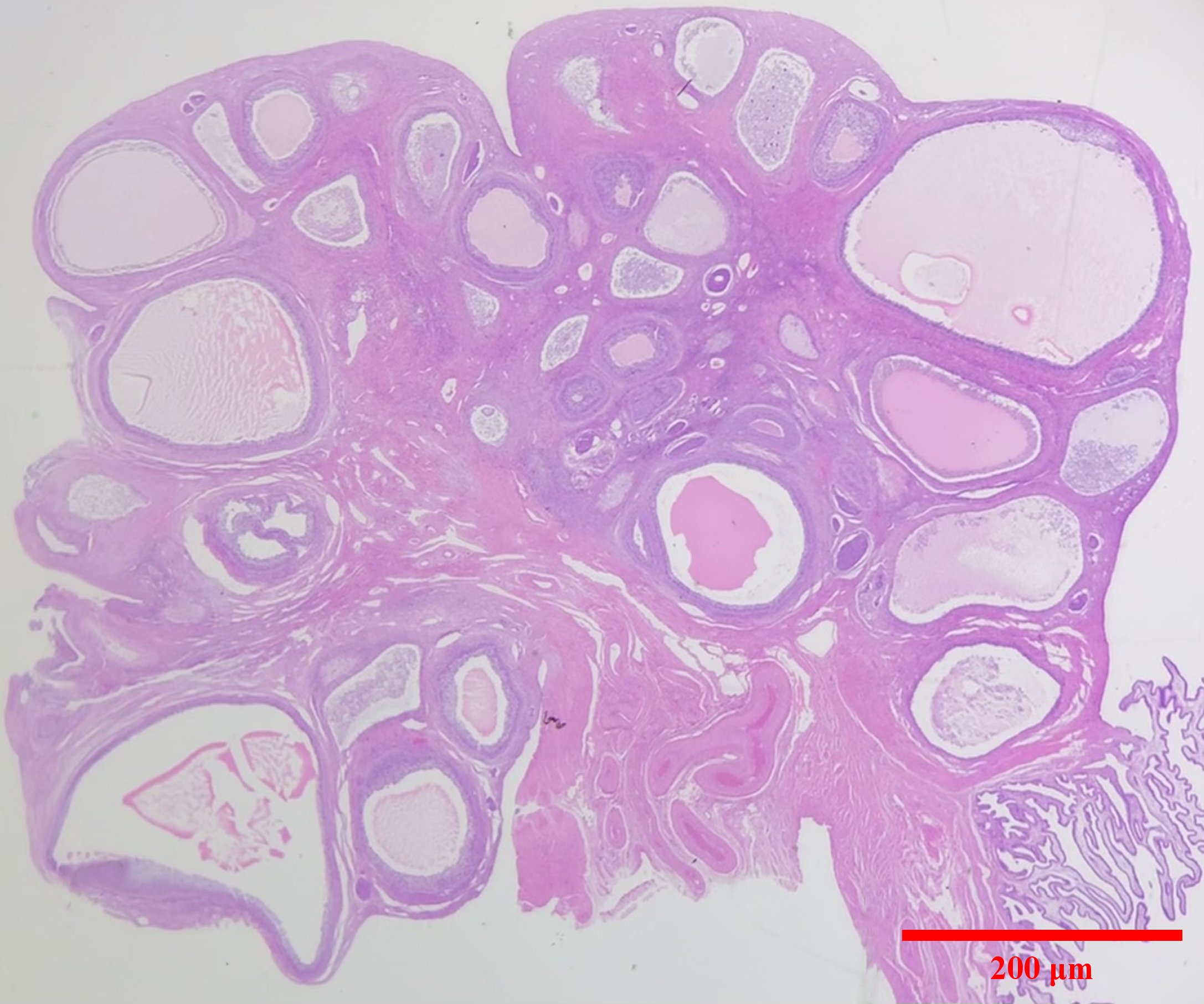

Supplement: Supplementary Figure 4 — H&E Staining Diagram of Ovarian Tissue in High-AMH Group. [file Image_4.jpeg]

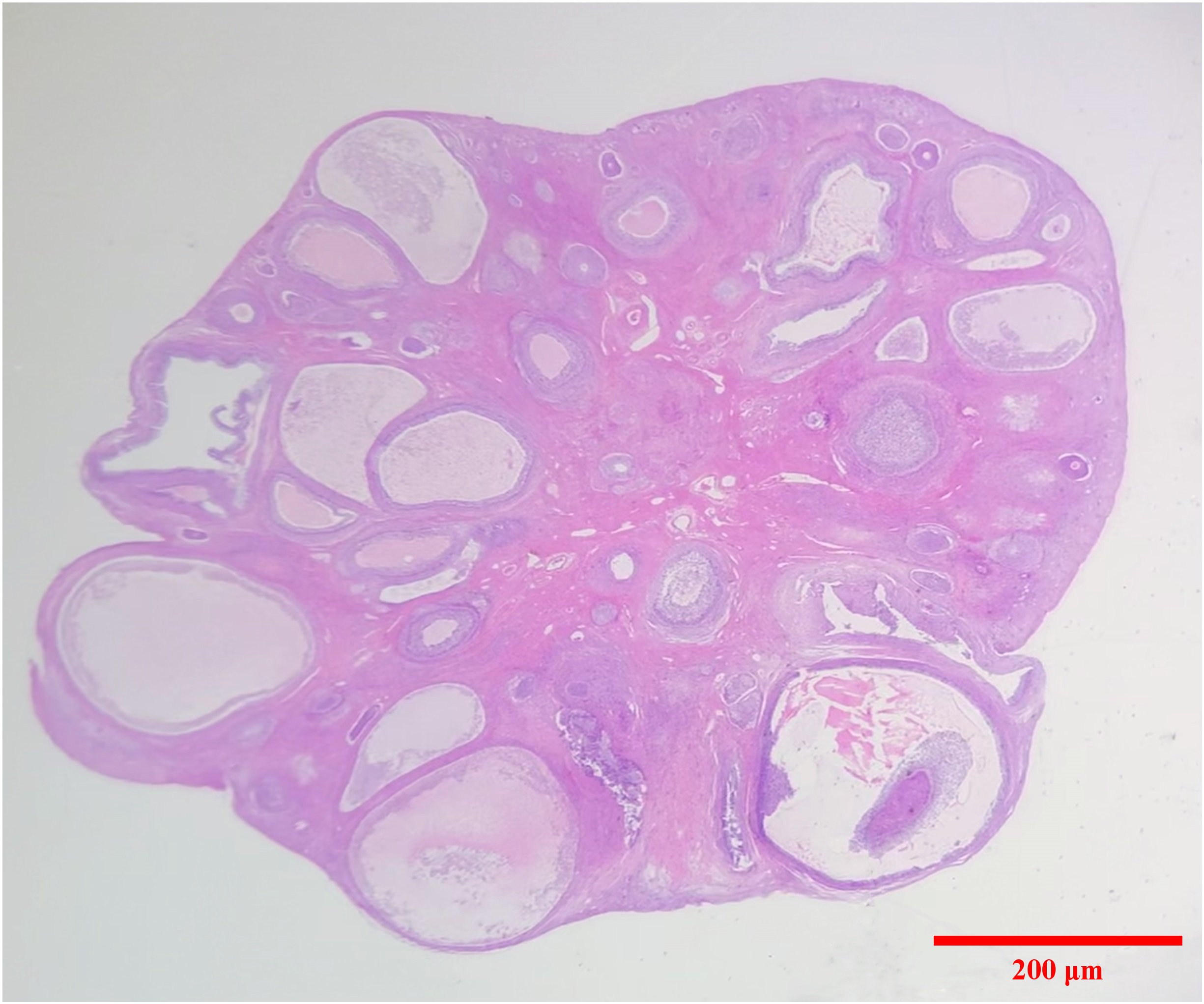

Supplement: Supplementary Figure 5 — H&E Staining Diagram of Ovarian Tissue in Low-AMH Group. [file Image_5.jpeg]

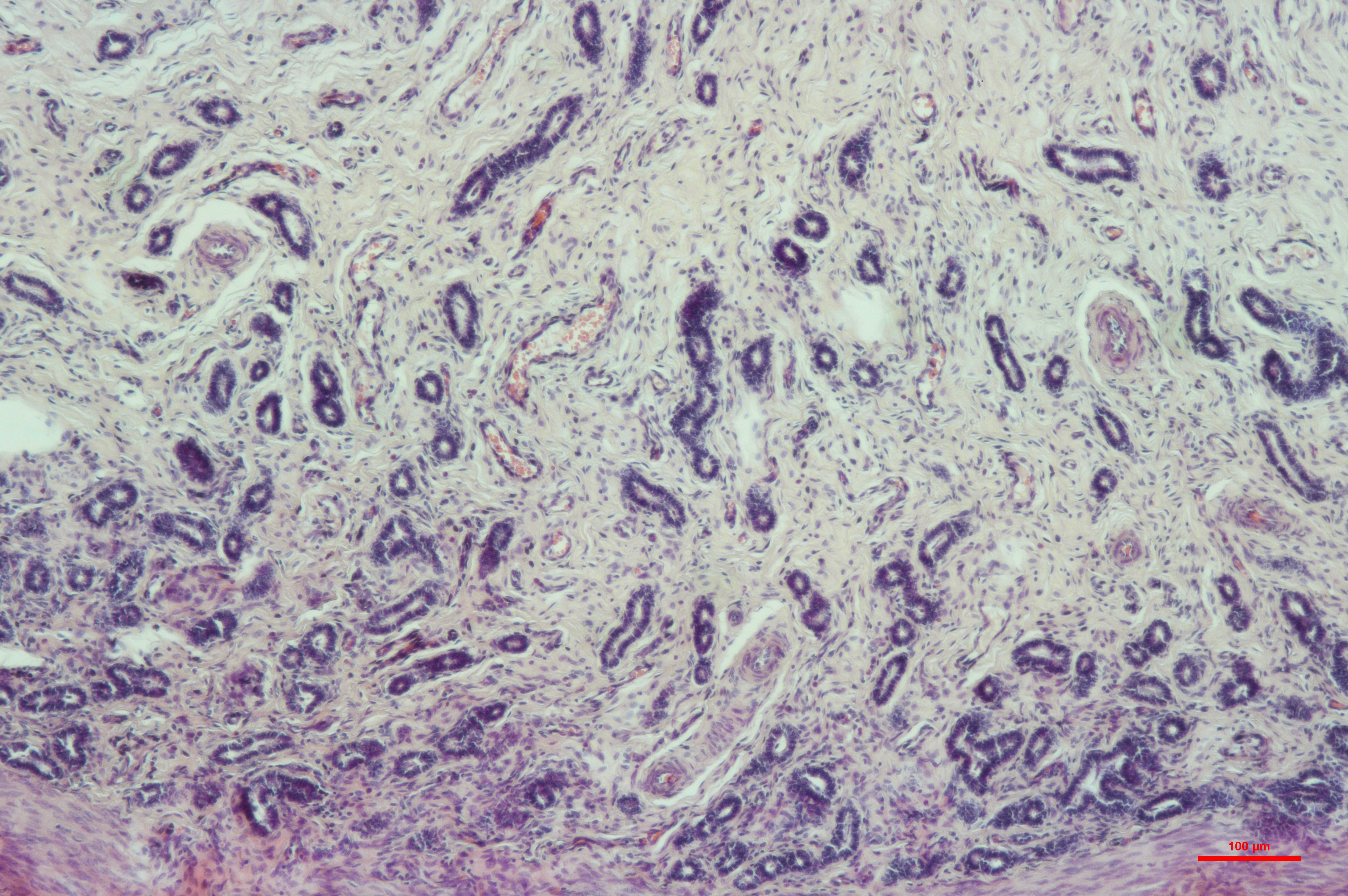

Supplement: Supplementary Figure 6 — H&E Staining Diagram of Uterine Tissue in High-AMH Group. [file Image_6.jpeg]

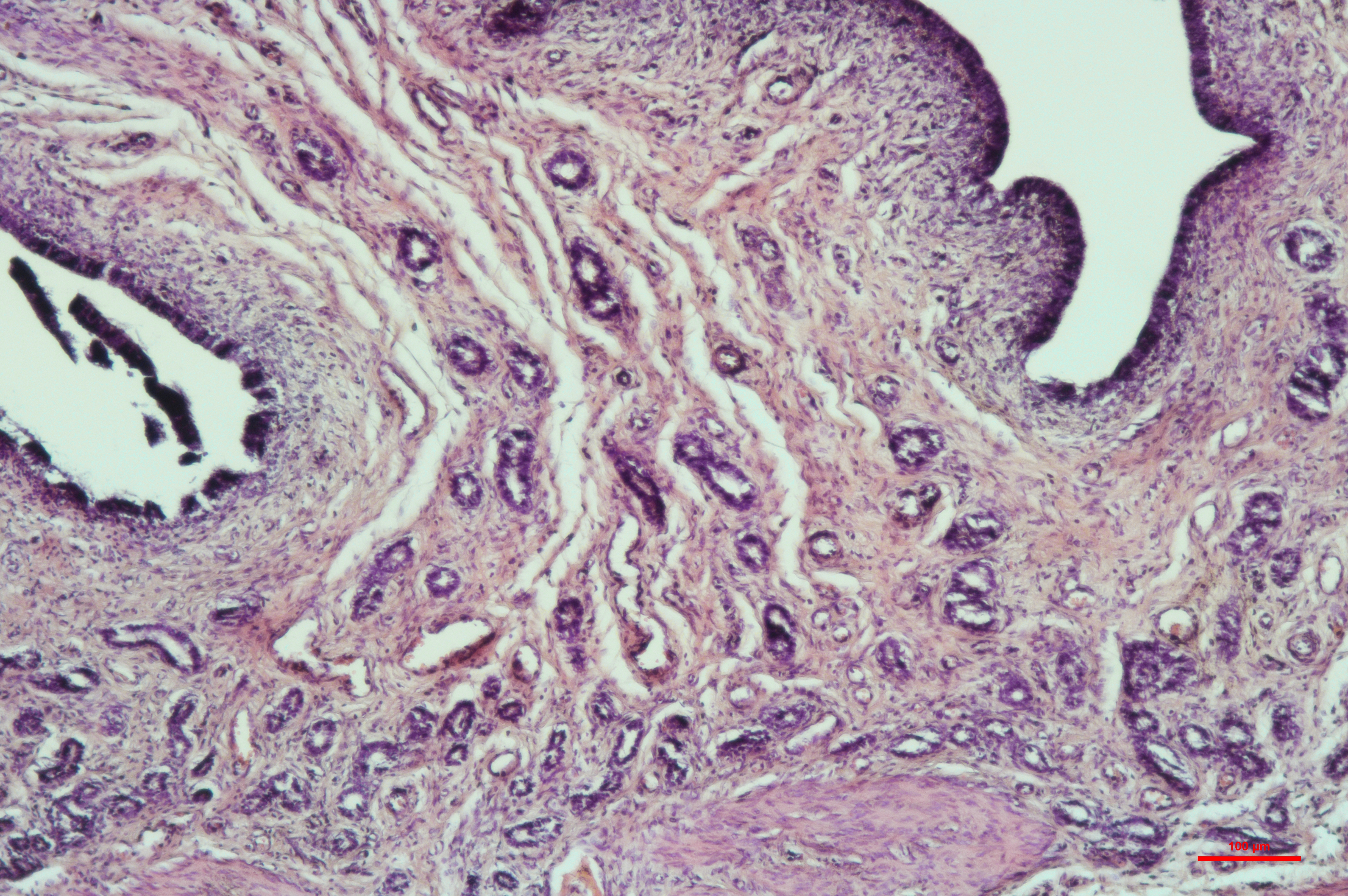

Supplement: Supplementary Figure 7 — H&E Staining Diagram of Uterine Tissue in Low-AMH Group. [file Image_7.jpeg]
